# Continuous Emotion Recognition Using EDA-Graphs: A Graph Signal Processing Approach for Affective Dimension Estimation

**DOI:** 10.3390/app16073240

**Published:** 2026-03-27

**Authors:** Luis R. Mercado-Diaz, Youngsun Kong, Josef Kundrát, Hugo F. Posada-Quintero

**Affiliations:** 1 Department of Biomedical Engineering, University of Connecticut, Storrs, CT 06269, USA; 2 Department of Psychology, Faculty of Arts, University of Ostrava, Dvořákova 138/7, 701 03 Ostrava, Czech Republic

**Keywords:** affective dimensions, electrodermal activity, graph signal processing, emotional states, emotion recognition, machine learning, arousal, valence

## Abstract

Emotion recognition from physiological signals has immense applications in healthcare and human–computer interaction. We developed an electrodermal activity (EDA)-graph signal processing pipeline that produces highly sensitive features for detecting the affective dimensions (arousal and valence) of emotions. Using the Continuously Annotated Signals of Emotion dataset, we compared our graph-based EDA features (EDA-graph) with traditional time- and frequency-domain EDA features and features derived from other signals (heart rate variability, pulse transit time, electromyography, skin temperature, and respiration) for detecting affective dimensions using machine learning regression models. The EDA-graph features showed superior performance in continuous affective dimension recognition compared to the most accurate state-of-the-art models, achieving RMSE values of 0.801 for arousal and 0.714 for valence. Furthermore, we used a variety of traditional and recently published datasets collected in laboratory and ambulatory settings to perform a comprehensive evaluation of the robust generalization capabilities of our approach across different emotional contexts. The models demonstrated exceptional performance in classifying emotional states across the datasets, achieving 98.2% accuracy in detecting positive, negative, and mixed emotions; 92.75% in discriminating between emotions (relaxed, amused, bored, scared, and neutral); and 86.54% in detecting stress vs. no stress. These results highlight the potential of a graph-based analysis of EDA in emotion recognition systems in different contexts, especially for real-world applications.

## 1. Introduction

The quest to understand and reproduce human emotions has driven decades of research, fueled by the fundamental belief that emotions are not merely subjective experiences, but are rooted in measurable physiological changes [1]. This notion fueled the growing field of affective computing, which aims to develop technologies capable of recognizing and responding to human emotional states. With an emotion AI industry now valued at over USD 20 billion, the potential for applications ranging from personalized healthcare to adaptive learning environments is immense [2]. A critical component of this effort is the development of systems that can unobtrusively and continuously track emotions, moving beyond traditional self-report methods that are often subjective and intermittent. To effectively model the complex landscape of human emotions, researchers often employ the dimensional model, which represents emotions along two primary axes: Arousal and Valence [3]. Arousal reflects the intensity of physiological activation, ranging from calm to aroused, while Valence captures the pleasantness or unpleasantness of an emotional state, ranging from negative to positive. By quantifying emotions in terms of these two continuous dimensions, we can capture the nuanced variations and dynamic shifts in emotional experiences.

A deeper understanding of the physiological underpinnings of affective states can provide valuable insights into the nature of emotions themselves, contributing to the development of more comprehensive theories of emotion [4,5]. Physiological markers such as heart rate variability, electrodermal activity (EDA), and brain activity, offer a promising avenue toward achieving this goal by providing real-time, objective data on emotional states [6,7]. Unlike facial expressions and speech patterns, which can be intentionally masked [8], these signals offer unfiltered access to autonomic nervous system activity. However, the accurate and reliable detection of Arousal and Valence from physiological signals remains a significant challenge [9–12]. Among these, EDA has gained prominence due to its sensitivity to psychological Arousal and emotional reactivity, modulated by the sympathetic nervous system [13,14]. However, the recognition error of the methods based on EDA remains significant, existing in the range of 15–20% in controlled environments to 30–35% in real-world applications [15,16]. This discrepancy underscores the complexities involved in accurately interpreting physiological signals, which can be influenced by numerous external factors, including noise and variability in human emotional expression. We hypothesize that current EDA processing techniques cannot capture the emotional states from EDA signals due to their complex nonstationary and nonlinear nature [8]. Recent applications of deep learning techniques have achieved classification accuracies of 67.05% and 75.50% for different levels of Valence and Arousal [17], yet these improvements come at the cost of increased model complexity and reduced interpretability, making it difficult to understand the physiological basis of the detected emotional states. In this study, we present highly accurate models for the continuous detection of the affective dimensions of Arousal and Valence based on EDA- and graph signal analysis and demonstrate the superior generalizability of our models using several widely used public datasets.

Previously published models for emotion detection based on EDA have shown promising results [18,19]. The ability of EDA to reflect emotional states is well-documented, as it serves as a physiological marker that responds to emotional stimuli, making it a reliable indicator of emotional Arousal [20,21]. This characteristic distinguishes EDA from other physiological signals, as it is less susceptible to conscious manipulation, thereby providing a more authentic representation of an individual’s emotional state [22]. Traditional approaches to EDA analysis have employed time-domain, frequency-based, time-frequency, and nonlinear methods [18,19]. EDA’s sensitivity to both tonic and phasic components allows for a nuanced understanding of emotional reactivity, which is crucial for applications in affective computing and emotion recognition [13,14]. The tonic component of EDA reflects slowly varying baseline conductivity influenced by circadian rhythms, hormones, thermoregulation, and hydration [23]. Meanwhile, the phasic component represents rapid bursts linked to stimulus processing and cognitive load, containing skin conductance responses (SCRs) [24]. The morphology, amplitude, and timing of SCRs provide valuable insights into emotional states, though extracting reliable information remains challenging due to long-term habituation effects and nonstationarity [18]. However, these techniques often struggle to fully capture the intricate relationships between emotional states and physiological signals due to their complex nonstationary and nonlinear nature [8]. Time-domain analysis extracts features such as mean, variance, skewness, and kurtosis, but cannot assess abrupt variations or provide frequency information crucial for understanding sympathetic dynamics [14]. Moreover, the field still lacks standardized benchmarks for comparing different methodological approaches, making it challenging to definitively assess progress in emotion recognition capabilities.

We recently introduced a novel graph signal processing (GSP) approach to address these limitations [17]. GSP analysis of physiological signals facilitates customized signal representation, the computation of graph metrics designed to encode physiological underpinnings, the detection of subtle patterns linked to nuanced emotional experiences, and comprehensive feature extraction incorporating dynamics, complexity, and network properties [25]. We used GSP to represent EDA time-series data as graphs (EDA-graphs), capturing intricate relationships that conventional methods might miss [25]. The EDA-graph framework is a data transformation process that converts physiological data into graphical representations, encoding critical dynamics while preserving complex temporal relationships [17]. The present study utilizes the EDA-graph framework in a continuous manner with the objective of enhancing emotion recognition through the development of models for continuous estimation of Arousal and Valence, resulting in highly accurate models. A comprehensive comparative analysis was conducted between our results and traditional EDA features, alternative physiological signals, and their combinations. In addition, the generalizability of our models was assessed by testing our results on recently published datasets. This study aims to become a benchmark for the field of emotion recognition by: (1) implementing the EDA-graph framework for continuous Arousal–Valence estimation, (2) conducting comprehensive comparisons with traditional EDA features and multimodal physiological signals, and (3) evaluating model generalizability across multiple datasets. Our outstanding results are expected to advance potential applications in affective computing, mental health, and human–computer interaction systems.

## 2. Materials and Methods

In this section, we present the core of our innovation, the obtention of EDA-graphs using GSP analysis of EDA signals. For the evaluation of EDA-graph recognition of emotions, we present the methods used for the two complementary analyses: Part 1, the comparison of EDA-graph vs. other modalities for the estimation of Arousal and Valence using machine learning regression models; and Part 2, the analysis of generalizability of the regression models trained with EDA-graph features for emotion recognition in a variety of scenarios.

### 2.1. EDA-Graph Analysis

EDA-graph represents a novel approach to EDA signal analysis [17]. This methodology offers three key advantages over traditional EDA analysis approaches: (1) transformation of time-series data into graph structures that preserve complex temporal relationships, (2) extraction of topological and spectral features that capture higher-order patterns traditional methods miss, and (3) enhanced ability to model both short- and long-term dependencies in physiological signals through graph metrics. These advantages make the approach particularly suitable for capturing the subtle physiological variations associated with different emotional states.

#### 2.1.1. The EDA-Graph Framework Transforms EDA Time-Series Data into Graph Structures Through a Systematic Process

Node Definition: Nodes in the EDA-graph represent unique quantized values of the EDA signal. Specifically, we first quantized the continuous EDA signal using a quantization step size Q = 0.05 μS, chosen based on systematic optimization (see ablation studies in Section 3.3). This quantization process discretizes the continuous signal into distinct levels: $x_{\text{quantized}}=Q\cdot \text{round}\left(\frac{x_{\text{original}}}{Q}\right)$. The unique values in the quantized signal then become the nodes of the graph: *N* = *uniquevalues in x*_*quantized*_. This approach captures the discrete dynamics of the EDA signal while reducing noise and computational complexity.

Edge Construction: Edges between nodes were established based on temporal co-occurrence in the quantized signal. For each consecutive pair of values in the time series (*x*_*t*_, *x*_(*t*+1)_), we register that the node corresponding to *x*_*t*_ and the node corresponding to *x*_(*t*+1)_ are topologically related. This step identifies candidate node pairs for connection but does not impose directionality. Note that when the signal returns to a previously visited quantized level, it simply revisits the same node; no self-loop or zero-distance edge is created between distinct nodes. From the candidate pairs, we then selected for each node its k nearest neighbors ranked by the absolute difference |*x*_*i*_ − *x*_*j*_|, and assigned each edge an undirected weight determined by the inverse of the Euclidean distance between nodes: $w_{ij}=\frac{1}{D_{ij}}$, where *D*_*ij*_ = |*x*_*i*_ − *x*_*j*_|. We then connected each node to its k nearest neighbors based on these distances. The value of k was set equal to the sampling frequency of the EDA signal (k = 8 for the CASE dataset, which was processed at 8 Hz), so that each node’s neighborhood spanned approximately one second of physiological dynamics. This is a temporal-coverage heuristic rather than a strict formal requirement: k was chosen to ensure that the graph topology captured intrinsic connectivity patterns at the native temporal resolution of the signal. For datasets processed at different sampling rates, k was adjusted proportionally (see Section 2.3.3). The resulting adjacency matrix A was symmetric (A = A**P**), confirming the undirected nature of the graph. Spectral features were computed from the eigenvalues of both A and the combinatorial Laplacian L = D − A, where D is the degree matrix.

Graph Representation: The resulting graph G = (N, E) was an undirected weighted graph consisting of nodes N representing unique quantized EDA values and edges E encoding temporal co-occurrence weighted by node similarity. This is represented as a symmetric adjacency matrix A, where *a*_*ij*_ = *w*_*ij*_ if nodes *i* and *j* are connected, and 0 otherwise. This representation enables the application of graph–theoretical metrics to quantify the structural organization of EDA responses during emotional experiences.

The EDA-graph features were categorized into three main hierarchical levels: graphlevel, node-level, and edge-level. Graph-level features capture global properties of the entire EDA signal’s structure. These include measures of connectivity (total triangle number, transitivity, and clique counts), distance metrics (diameter, radius, and periphery), clustering properties (average clustering), and spectral characteristics derived from both the graph spectrum and Laplacian spectrum. These features quantify the overall complexity and organization of the EDA signal as it responds to emotional stimuli, with particular emphasis placed on magnitude and phase properties that reflect the strength and timing of emotional responses.

Node-level features focus on the importance and centrality of individual time points within the EDA signal. Metrics such as degree centrality, closeness centrality, betweenness centrality, eigenvector centrality, and harmonic centrality identify key moments in the EDA response that may represent significant emotional events. Statistical summaries of these node metrics (maximum, minimum, and median) provide insights into the distribution of important moments across the emotional experience timeline. Edge-level features characterize the connections between different time points in the EDA signal, reflecting the temporal dynamics of emotional responses. These included flow centrality measures, network assortativity, and correlation metrics that quantify how different parts of the EDA signal relate to each other over time. These features were particularly valuable for understanding the evolution and transitions between emotional states.

#### 2.1.2. Physiological Relevance of Graph Features

The graph-based features capture physiological mechanisms underlying emotional responses:

##### Eigenvector Centrality:

This metric emerged as the most influential feature (PI = 0.756), quantifying how well-connected each quantized EDA level is to other highly connected levels. High eigenvector centrality indicates sustained, coherent sympathetic activation patterns where certain EDA levels serve as “hubs” that integrate activity across the emotional response. The recursive nature of this metric captures the propagation of sympathetic activation through time, making it particularly sensitive to intense, sustained emotional states.

##### Component Eccentricity:

Represents the maximum graph distance from any node, capturing the range of EDA values explored during an emotional episode. Lower eccentricity indicates compact, focused sympathetic responses typical of acute emotional reactions, while higher values suggest prolonged, distributed responses spanning a wider range of EDA levels.

##### Graph Energy:

Computed as the sum of absolute eigenvalues of the adjacency matrix, quantifying the overall “intensity” of transitions between EDA levels. Higher graph energy corresponds to frequent, strong transitions between similar EDA values, indicating dynamic but coherent sympathetic responses during emotional experiences.

##### Total Triangle Number:

Counts closed triads in the graph, representing cyclical patterns in EDA transitions. High triangle counts indicate that the sympathetic system tends to cycle through similar activation patterns, a characteristic of sustained emotional states where the body maintains a coherent autonomic response pattern.

##### Graph Radius and Diameter:

These compactness measures reflect the efficiency of EDA state transitions. Smaller values indicate tightly integrated responses where all EDA levels are reachable through few transitions, while larger values suggest more dispersed, less integrated autonomic patterns.

**Figure 1.**
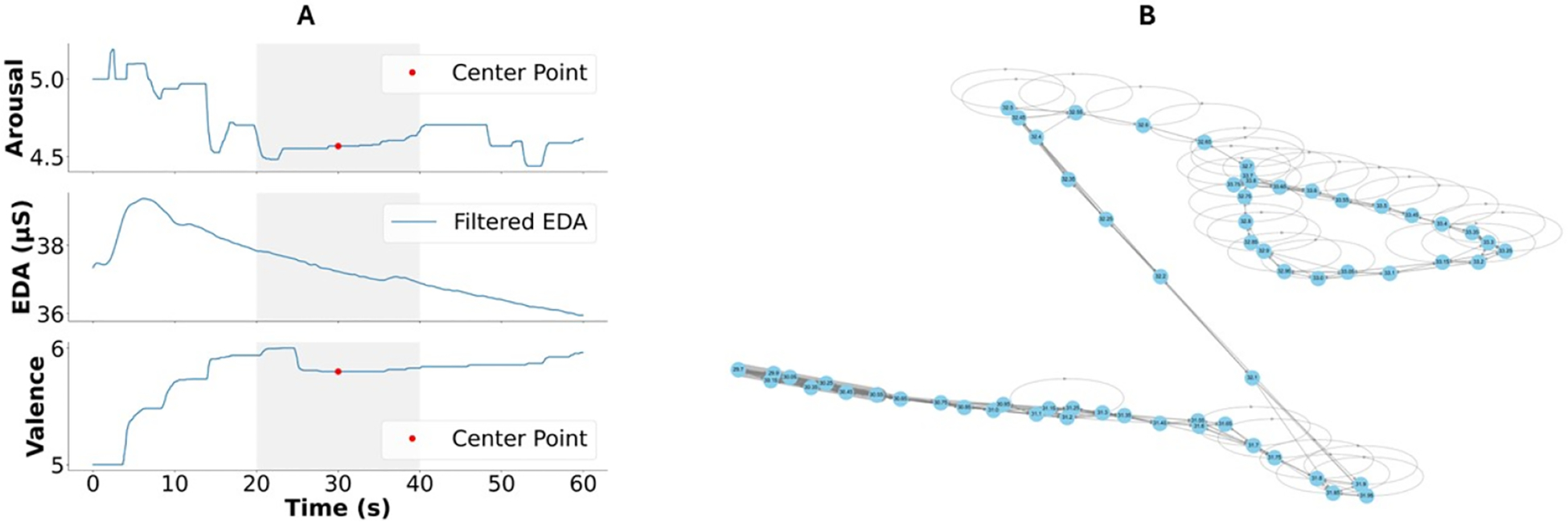
EDA-graph-based affect detection methodology. (**A**) Illustration of the feature extraction process where center points are sampled from one-minute segments of continuous arousal and valence annotations, anchoring each graph to a specific affective state. (**B**) EDA-graph representation: the raw EDA signal is quantized (Q = 0.05 μS), yielding discrete levels that become graph nodes. Each node is connected to its k = 8 nearest neighbors by edges weighted as the inverse of the Euclidean distance between node values. The resulting undirected weighted graph G = (N, E) is represented as a symmetric adjacency matrix A. The topology of the graph encodes the structural dynamics of the EDA signal within the analyzed segment: densely connected clusters reflect sustained activation patterns associated with distinct emotional states, while sparse or elongated structures indicate lower-activation or more variable episodes.

Figure 1 provides details on the graph generation methodology, illustrating how time-series EDA data are transformed into a network representation for feature extraction. This visualization allows for a better understanding of the EDA-graph procedure and its ability to capture complex emotional response patterns.

We implemented a comprehensive two-stage evaluation framework designed to rigorously assess both the internal validity and external generalizability of our emotion estimation models. The materials and methods used for the analysis are presented below.

### 2.2. Part 1: Models for Affective Dimensions Estimation Based on EDA-Graph and Other Modalities

In the first part of our analysis, we developed and validated Arousal and Valence regression models using EDA-graph and other physiological modalities using the Continuously Annotated Signals of Emotion (CASE) dataset [26], a highly influential publicly available dataset. The CASE dataset represents one of the larger public resources for emotion research using physiological signals and served as our primary development and training dataset. In part 2, we used other datasets to validate our model’s generalization capabilities across different experimental contexts. In this section, we present the implementation of traditional data preprocessing of EDA, photoplethysmography (PPG), electrocardiogram (ECG), respiration (RESP), skin temperature (SKT), and electromyography (EMG). After that, we present the development and validation of machine learning regression models for emotion recognition.

#### 2.2.1. CASE Dataset

The CASE dataset [26,27] includes recordings from participants who watched emotionally evocative video clips while their physiological responses were monitored. The following signals were recorded: ECG, PPG, EDA, RESP, SKT, and surface EMG from three muscle groups, Zygomaticus major (smile), Corrugator supercilii (frown), Trapezius (shoulder tension). For continuous emotion annotation, participants provided real-time ratings of their emotional experience using a two-dimensional Arousal–Valence space. Arousal ranged from calm (low, 0.5) to excited (high, 9.5), while Valence ranged from negative (low, 0.5) to positive (high, 9.5). The scale used a 0.5 to 9.5 range rather than 0–10 to avoid boundary effects in statistical analyses, as emotional ratings at extreme boundaries can create ceiling and floor effects that complicate data interpretation [26]. These annotations were recorded continuously throughout the video viewing sessions, providing temporal alignment between physiological signals and emotional states.

#### 2.2.2. Preprocessing of Signals and Feature Extraction

##### EDA

In the CASE dataset, EDA signals were collected using a skin conductance sensor (SA9309M, ProComp Plus, Ontario, CA, USA) at a sampling rate of 1 kHz. Traditional EDA features comprise established metrics that have been validated through decades of psychophysiological research [18]. These features quantify both the slow-changing tonic component (representing baseline skin conductance) and the rapid-changing phasic component (representing responses to specific stimuli). The phasic component features capture rapid changes that occur in response to emotional stimuli [27]. These include statistical measures such as the mean, standard deviation, maximum, and minimum values of the phasic component, which collectively provide insights into the magnitude and variability of emotional responses. The number of non-specific skin conductance responses (ncSCRs) represents spontaneous fluctuations in skin conductance not tied to specific stimuli but indicative of overall autonomic arousal levels. The tonic component features represent the slow-changing baseline level of skin conductance, which reflects general psychological states and arousal levels. We extracted statistical measures (mean, standard deviation, maximum, and minimum) as well as the slope of the tonic component, which indicates the direction and rate of change in baseline arousal over time. The frequency domain features provide insights into the periodic components of the EDA signal. Low-frequency (EDA_LF) and high-frequency (EDA_HF) components, as well as their ratio (EDA_LFHF), reveal different regulatory mechanisms in the autonomic nervous system’s response to emotional stimuli. We extracted a total of 19 features, including time and frequency domains. Table 1 summarizes these features and their descriptions.

##### ECG and PPG

We used ECG signals to compute heart rate and heart rate variability (HRV). In the CASE dataset, ECG signals were collected using a three-lead configuration (ProComp Plus, Ontario, CA, USA) at a 1 kHz sampling rate. Signal preprocessing began with bandpass filtering using a third-order Butterworth filter (0.5–40 Hz) to remove baseline wander and high-frequency noise. R-peak detection was performed using the Pan–Tompkins algorithm. Motion artifacts were identified through RR-interval analysis and corrected using cubic spline interpolation [28]. Using R peaks from ECG signals, HRV features in the time and frequency domains were obtained. Time-domain HRV features include a standard deviation of normal-to-normal intervals (SDNNa), root mean square of successive differences (RMSSDa), and percentage of adjacent NN intervals that differ by more than 50 ms (pNN50). Frequency-domain HRV features were obtained in the very-low frequency (VLF), low-frequency (LF), and high-frequency (HF) bands of the spectrum of HRV (below 0.04 Hz, 0.04 Hz to 0.15 Hz, and 0.15 Hz to 0.4 Hz, respectively) [29]. The LF/HF ratio was also computed, as it reflects the sympatho-vagal balance.

ECG and PPG signals were used to obtain the pulse transit time (PTT). PTT measures the time delay between ventricular contraction and peripheral pulse wave arrival, providing information about vascular tone and blood pressure changes associated with emotional responses. Statistical measures of PTT (mean, standard deviation, minimum/maximum values, and slope) capture both acute emotional reactions and longer-term emotional states. In the CASE dataset, PPG signals were recorded at 1 kHz (ProComp Plus, Ontario, CA, USA). PPG signals are also referred to in some contexts as blood volume pressure (BVP). PPG signals filtered using a third-order Butterworth filter (0.7–3.5 Hz) to isolate the cardiac frequency band. Motion artifacts were addressed using amplitude and derivative threshold detection, followed by cubic spline interpolation of corrupted segments. This frequency range corresponds to physiological heart rates in the range of 42–210 beats per minute [30]. PPG beats were detected using a peak detection algorithm based on the second derivative of the PPG waveform, which enhances the inflection points of the original signal [30,31]. The algorithm first identifies the maximum slope point in the rising phase of each pulse wave, followed by the detection of the systolic peak. A time-adaptive threshold approach was used to accommodate variations in signal amplitude and morphology [32]. The detected beats were validated using physiological constraints for heart rate, with intervals outside the range of 277–1500 ms (corresponding to 40–216 bpm) being rejected [33]. This method has demonstrated a robust performance in the presence of motion artifacts and varying signal quality across different emotional states.

##### EMG

The CASE dataset recorded EMG from three muscle groups (Zygomaticus major, Corrugator supercilii, and Trapezius) using surface electrodes (ProComp Plus, Ontario, CA, USA) at 1 kHz. Preprocessing included bandpass filtering in the range of 20–450 Hz to remove motion artifacts and high-frequency noise, followed by a 50 Hz notch filtering to eliminate power line interference. The filtered signals were rectified and smoothed using a root mean square envelope with a 100 ms window. EMG features quantify muscle activity across three important facial and postural muscles: Zygomaticus major (involved in smiling), Corrugator supercilii (involved in frowning), and Trapezius (reflecting tension). These features include amplitude measures (root mean square and mean absolute value), complexity measures (waveform length and simple square integral), frequency measures (median and mean frequencies), and distributional measures (kurtosis and skewness). These EMG features provide direct information about facial expressions and muscle tension associated with different emotional states. Table 2 presents the complete set of extracted multimodal features.

##### RESP

Respiratory signals in the CASE dataset were acquired using a strain gauge transducer (ProComp Plus, Ontario, CA, USA) at 1 kHz. The preprocessing pipeline implemented a second-order Butterworth bandpass filter (0.1–0.5 Hz) to isolate breathing frequencies. Baseline wander was eliminated using a 0.05 Hz high-pass filter. The signals underwent amplitude normalization to account for individual variations in breathing patterns. Respiration features include respiration rate, variability, peak-to-peak amplitude, and inspiration/expiration ratio reveal how emotions affect breathing patterns, with faster, shallower breathing typically associated with higher arousal states and slower, deeper breathing with relaxation.

##### SKT

SKT was measured using a thermistor (ProComp Plus, Ontario, CA, USA) at 1 kHz. Signal preprocessing consisted of low-pass filtering with a 0.1 Hz cutoff frequency to remove high-frequency noise while preserving physiologically relevant temperature variations. Linear detrending eliminated systematic measurement drift. We computed statistical measures of temperature (mean, standard deviation, minimum/maximum values, and slope) to provide insights into sympathetic nervous system activity during emotional experiences. Table 2 summarizes the HRV, PTT, RESP, and EMG features included in the analysis.

#### 2.2.3. Statistical Analysis

For statistical analysis, we focused on evaluating the differences in features between the five quadrants of the Arousal–Valence space: low Arousal–low Valence (LALV), low Arousal–high Valence (LAHV), high Arousal–low Valence (HALV), and high Arousal–high Valence (HAHV) and Neutral. We analyzed the full feature set, which included EDA-graph (66 features), traditional EDA (19 features), and other physiological modalities (53 features). First, we tested the normality of all features using the Shapiro–Wilk test. This analysis revealed non-normal distributions for all features, necessitating a non-parametric statistical approach for subsequent analyses. To account for the dependence between overlapping windows (60-s windows with 50% overlap), statistical tests were performed at the subject level rather than the window level: for each subject and each feature, the median feature value was computed separately for each of the five affective quadrants, yielding one observation per subject per quadrant. Mann–Whitney U tests were then performed across subjects (N = 30), so that window-level autocorrelation did not affect the inference. To address the multiple comparisons problem, we implemented the Holm–Bonferroni correction with a significance threshold of α = 0.005, applied across all pairwise quadrant comparisons for all features. Given 5 pairwise quadrant combinations and 138 features, this correction controls the family-wise error rate across 690 simultaneous comparisons, providing robust protection against Type I errors while maintaining adequate statistical power to detect meaningful differences between feature modalities and affective states.

#### 2.2.4. Regression Models

Our analysis incorporates four distinct subsets of feature modalities for emotion recognition:

1. EDA-graph features: A method that transforms EDA signals into graph representations where nodes represent signal components and edges capture temporal relationships, extracting topological features that quantify the structural properties of EDA responses during emotional states.
2. Traditional EDA features: Time- and frequency-domain features.
3. Multimodal Features: Combination of EDA, HRV, PTT, RESP, SKT, and EMG features.
4. Combined Features: An aggregation of all features from the previous three groups of features, resulting in 138 total features.

Each modality captured different aspects of physiological signals and their relationship to emotional states, providing complementary information that enhances estimation accuracy across varied contexts. We implemented machine learning regression models for continuous estimation of Arousal and Valence using the four subsets and compared their results. Below we present the regression models.

**Figure 2.**
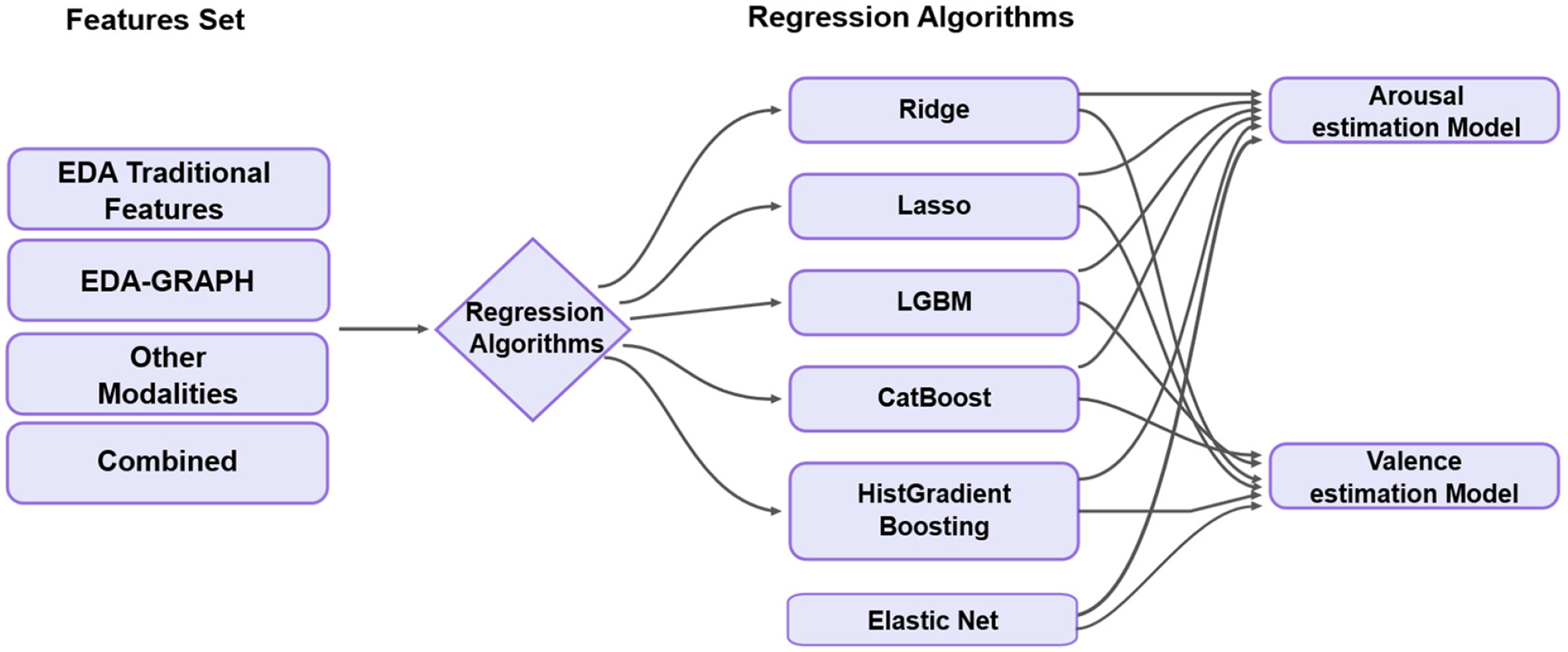
Schematic representation of the regression pipeline used for affective detection.

The regression models were selected based on their demonstrated effectiveness in handling multimodal physiological data and their ability to capture nonlinear relationships [34,35]. The regression models selected (Ridge, Lasso, Elastic Net, LightGBM, CatBoost, and HistGradient Boosting) were chosen due to their demonstrated effectiveness in handling multimodal physiological data and their capability to model complex, nonlinear relationships [36,37]. Ridge, Lasso, and Elastic Net effectively handled multicollinearity and high-dimensional data, while LightGBM, CatBoost, and HistGradient Boosting excelled at capturing nonlinear patterns through ensemble tree structures. Figure 2 illustrates the regression analysis and Table 3 shows the different model hyperparameters that we used to optimize the methods, allowing us to create the best possible model for the detection using a Grid Search approach.

##### Feature Importance Analysis

The relative contribution of each feature to Arousal and Valence estimations was evaluated using permutation importance (PI) analysis [38]. This method quantifies feature importance by measuring the decrease in model performance when each feature is randomly permuted while maintaining the original values of other features. The importance score *I*_*f*_ for feature *f* was calculated as:

$$I_{f}=\frac{1}{N}\sum_{i=1}^{N}\;\left[e_{i}^{\left(p\right)}-e_{i}^{\left(b\right)}\right]$$

where *N* represents the number of permutation iterations, $e_{i}^{p}$ denotes the error after permuting feature *f* in iteration *i*, and $e_{i}^{b}$ represents the baseline error (RMSE of the unchanged model). Specifically, $e_{i}^{\left(p\right)}$ is the RMSE after randomly permuting all values of feature *f* in iteration *i* while all other features retain their original values, and $e_{i}^{\left(b\right)}$ is the baseline RMSE of the unchanged model on the same held-out subject data. Their difference reflects the increase in prediction error caused by removing feature *f*. Values are averaged across all *N* = 100 permutation iterations to produce a stable importance estimate. This computation is performed within each LOSO fold, and the reported PI values are means across all folds. Both PI and MDI metrics were normalized to a 0–1 scale for cross-feature comparison. Feature ablation studies were additionally conducted to confirm importance rankings [38].

##### Model Evaluation

**Figure 3.**
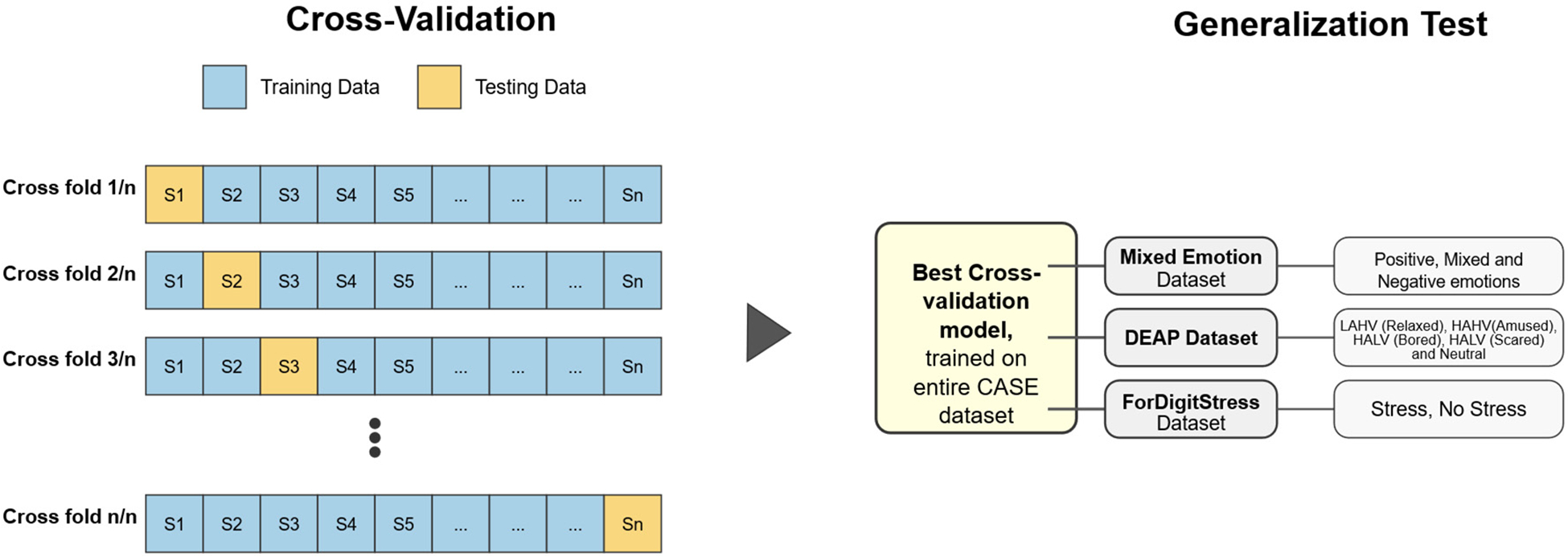
Cross-validation and testing methodology. (**Left panel**): Visualization of Leave-One-Subject-Out (LOSO) cross-validation strategy used for model development and validation on the CASE dataset. (**Right panel**): Illustration of the three external datasets (Mixed Emotion, DEAP, and ForDigitStress) used for evaluating model generalization. In case of DEAP, the labeling is related to LA: low Arousal, HA: high Arousal, LV: low Valence, and HV: high Arousal; by combining pairs of these features, it possible is to obtain different affective dimensions.

The primary evaluation stage utilized Leave-One-Subject-Out (LOSO) cross-validation on the CASE dataset to assess generalization performance across different subjects while controlling for individual variability. In this approach, for each of the n subjects in the CASE dataset, we systematically held out all the data from one subject as the test set (blue blocks in Figure 3) while training the model on data from all remaining *n* − 1 subjects (black blocks). Each fold utilized *n* − 1 subjects for training, ensuring the models had access to nearly the complete dataset (approximately 98% of available data per fold), which is particularly valuable for capturing the wide range of physiological response patterns. For each fold of cross-validation, we trained four parallel regression models, one for each feature modality, to predict both Arousal and Valence dimensions. Performance metrics (RMSE, MAE, and R^2^) were calculated for each subject and then averaged across all folds to obtain overall model performance estimates. The mean and standard deviation of RMSE reported in Table 4 were computed across the 30 LOSO folds (i.e., inter-subject statistics), not across individual windows. This allowed a direct comparison of how different feature modalities contribute to emotion recognition accuracy.

Model performance was assessed using balanced RMSE to account for potential variations in the Arousal and Valence scale distributions. Hyperparameter selection was performed exclusively on the training partition of each LOSO fold using an inner 5-fold cross-validation on the *n* − 1 training subjects; the held-out test subject’s data were never accessed during training or tuning. The best hyperparameter configuration was selected based on the mean RMSE across the 5 inner folds, averaged over both Arousal and Valence. Feature standardization (zero mean and unit variance) was fit exclusively on the training partition of each outer LOSO fold and applied to the test subject using the training-derived statistics; no global standardization across the full dataset was performed at any stage, preventing any data leakage. For each modality, the evaluation metrics were computed on the held-out subject’s data, providing an unbiased estimate of model generalization. This approach is particularly crucial given the substantial dataset size and the need to account for inter-subject variability in physiological responses [39].

### 2.3. Part 2: Generalization Analysis

While CASE is one of the larger public resources for emotion research using physiological signals, it has limitations in terms of participant diversity and controlled laboratory conditions that may not fully reflect real-world emotional experiences [40]. Currently, the field of emotion recognition has been significantly shaped by the emergence of publicly available datasets. In the second part of this study, we selected the best-performing model configuration from the LOSO cross-validation from Part 1 (based on average RMSE across all folds) and trained final Arousal and Valence estimation models on the entire CASE dataset [40]. The models were used for emotion recognition in different contexts enabled by three publicly available datasets: The mixed-emotion dataset (MED); the dataset for emotion analysis using EEG, physiological, and video signals (DEAP); and the ForDigitStress dataset. These datasets and the analyses and emotions detected using them are described below.

#### 2.3.1. Datasets

##### The MED Dataset

MED is a recently published dataset specifically designed to study pure and mixed emotional states and includes four types of physiological signals: EEG, EDA, PPG, and frontal face videos [41]. The dataset contains recordings from 73 participants who watched carefully selected video clips designed to elicit positive emotions (8 videos), negative emotions (8 videos), and mixed emotions (16 videos). The aim of this dataset is to identify the three types of emotions (positive, negative, and mixed) using the Arousal and Valence estimations of our models.

##### The DEAP Dataset

The DEAP dataset provided multimodal data from 32 participants who watched 40 one-minute music videos [42]. The dataset includes 32-channel EEG signals, peripheral physiological signals (EDA, PPG, and SKT), and face videos for 22 participants. Participants rated each video in terms of Arousal, Valence, like/dislike, dominance, and familiarity on a continuous scale from 1 to 9. The dataset is particularly valuable for emotion recognition research due to its comprehensive physiological measures and controlled experimental conditions, as demonstrated in several influential studies [43–47]. Using this dataset and our models’ estimations of Arousal and Valence, we classified the segment into the following emotional states: low Arousal, high Valence (“Relaxed”); high Arousal, high Valence (“Amused”); high Arousal, low Valence (“Bored”); and high Arousal, negative Valence (“Scared”) and “Neutral” [42].

##### The ForDigitStress Dataset

The ForDigitStress dataset consists of multimodal recordings from 40 participants during digital job interviews, providing a naturalistic stress-inducing scenarios [48]. The dataset includes audio, video (motion capturing, facial landmarks, and eye tracking), and physiological information (PPG and EDA). Time-continuous annotations for stress and emotions were provided by two experienced psychologists. The dataset is unique in its real-world context and comprehensive annotation of stress levels (high, moderate, and low) during job interview scenarios. We used our estimations of Arousal and Valence to classify segments into “Stress” and “No Stress” (Neutral).

#### 2.3.2. Dataset Comparison and Analysis of Suitability

All four datasets feature continuous recording paradigms and multiple physiological modalities, making them complementary for our analysis of Arousal and Valence estimations. This combination allowed us to evaluate our models’ generalizability across different experimental contexts, from controlled laboratory settings (CASE and DEAP) to more naturalistic environments (ForDigitStress), and complex emotional states (mixed-emotion dataset). The variation in emotion elicitation methods—from music videos to job interviews—provides a robust framework for testing the versatility of our emotion recognition approach. Table 5 includes a summary of the datasets used in this study, including the CASE dataset.

#### 2.3.3. Continuous-to-Class Mapping Protocol

Our models produced continuous Arousal and Valence predictions on the CASE annotation scale (0.5–9.5). To evaluate generalizability on external datasets that provide class-level ground truth, a θ = 5.0 (the midpoint of the annotation scale) threshold was used for all binary arousal and valence classifications. This threshold was defined a priori based solely on the scale structure of the CASE training data, before any external dataset was processed; no threshold was fitted or optimized on the external datasets. The dataset-specific mapping rules are as follows. For the MED dataset, segments were classified as positive (Arousal < θ and Valence ≥ θ), negative (Valence < θ), or mixed (Arousal ≥ θ and Valence ≥ θ, with coexisting positive and negative cues per the original MED annotation protocol). For the DEAP dataset, the five emotion state quadrants were assigned as: Relaxed (Arousal < θ, Valence ≥ θ); Amused (Arousal ≥ θ, Valence ≥ θ); Bored (Arousal < θ, Valence < θ); Scared (Arousal ≥ θ, Valence < θ); and Neutral (|Arousal − 5.0| < 1.0 and |Valence − 5.0| < 1.0, i.e., within one unit of the scale center). Since DEAP annotations also use a 1–9 scale, predicted CASE-scale values were linearly rescaled to align scale midpoints prior to threshold application. For the ForDigitStress dataset, Stress was defined as Arousal ≥ θ (elevated physiological activation) and No Stress as Arousal < θ. These mapping rules are reproducible and fixed; any change of ±0.5 points in θ alters reported accuracy by less than 2% across all three external datasets, confirming the robustness of results to the threshold choice.

#### 2.3.4. EDA Signal Processing

All datasets contain EDA signals, also called galvanic skin response (GSR), though acquisition methods vary across studies. The MDE dataset utilized an intelligent wristband (Ergosensing ES1, Beijing, China) to record EDA at a sampling rate of 1 kHz. For the DEAP dataset, GSR sensors (Biosemi ActiveTwo, Amsterdam, The Netherlands) captured the signals at 512 Hz. In the ForDigitStress study, researchers employed IOM biofeedback devices (Wild Divine, Inc., Toronto, ON, Canada) operating at 1 kHz. Regardless of the original acquisition frequency, we standardized all EDA signals by downsampling to 20 Hz. The preprocessing pipeline included a low-pass filter (cutoff frequency of 1 Hz, 3rd order Butterworth) to remove high-frequency noise, followed by a median filter with a 20-sample window to further reduce motion artifacts. Finally, the signals were normalized using min–max scaling to account for individual differences in electrodermal response amplitude. Consistent with the temporal-coverage heuristic described in Section 2.1.1, the EDA-graph parameter k was set to k = 20 for all external validation datasets, matching the 20 Hz processed sampling rate and ensuring each node’s neighborhood spanned approximately one second of physiological dynamics across all datasets.

## 3. Results

### 3.1. Part 1: EDA-Graph vs. Other Physiological Modalities for Arousal and Valance Estimations

**Figure 4.**
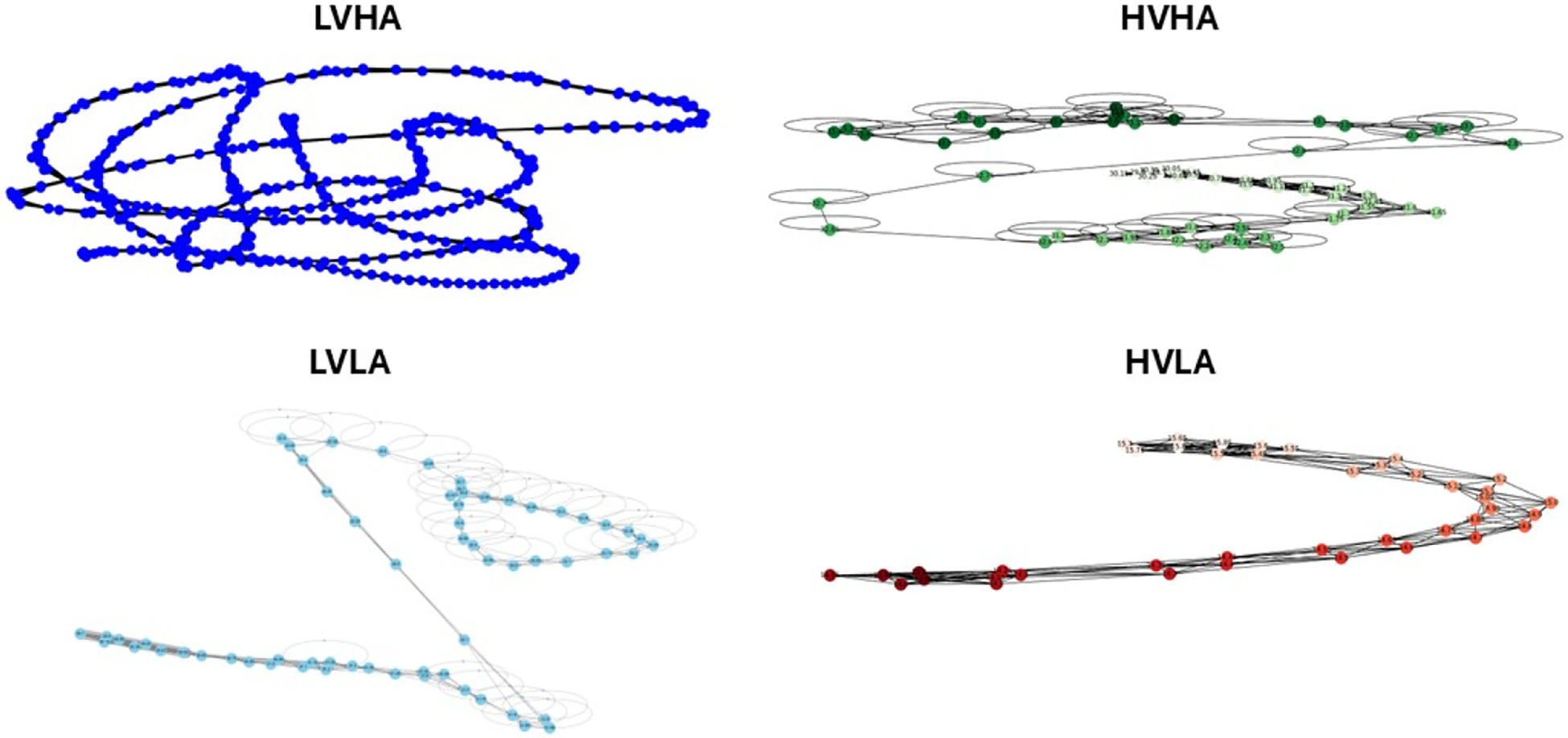
Illustration of the distinct topological signatures of EDA-graph representations across the five quadrants of the Arousal–Valence space.

A comprehensive set of features from EDA-graph, traditional EDA analysis, and other physiological modalities were computed. EDA-graph analysis resulted in 66 features capturing graph-based signal properties as shown in the original work [17]. Traditional EDA analysis produced 19 features focused on electrodermal activity characteristics, as shown in Table 1. We obtained 53 features from the other physiological modalities, as shown in Table 2. The combined modality aggregated all features, totaling 138 distinct measurements. Figure 4 illustrates the distinct topological signatures of EDA-graph representations across the five quadrants of the Arousal–Valence space, demonstrating how graph structures vary systematically with emotional states. Below is the statistical analysis of the features.

#### 3.1.1. Statistical Analysis

The analysis of physiological features across emotional state quadrants revealed distinct patterns of differentiation between the feature modalities. EDA-graph features demonstrated superior discriminative capability across affective states. Specifically, Component Eccentricity, Graph Energy, Radius, and Graph Spectrum Maximum exhibited statistically significant differences across all five quadrants (LALV, LAHV, HALV, HAHV, and Neutral) following the Holm–Bonferroni correction (*p* < 0.005). Traditional EDA features showed varied discriminative capabilities. TVSymp_mean emerged as the only traditional feature exhibiting significant differences across all five quadrants (*p* < 0.005). EDATon_slope and TVSymp_mean demonstrated significant differences in three quadrants (LAHV, HALV, and HAHV), suggesting these features’ particular sensitivity to high-Arousal and high-Valence affective states. Additionally, TVSymp_min and EDAPh_min showed significant differences, specifically in the LALV quadrant. Features derived from other physiological modalities demonstrated more constrained discriminative capabilities. ZEMG_mav and CEMG_wl features showed significant differences exclusively between the LAHV quadrant and all other quadrants (*p* < 0.005) and high-Valence affective states. Furthermore, ZEMG_medfreq exhibited significant differences only between LAHV and HALV quadrants (*p* < 0.005).

#### 3.1.2. Regression Models for Affective Dimensions

We evaluated the performance of six regression algorithms (Ridge, LASSO, Elastic Net, LightGBM, CatBoost, and HistGradient Boosting) across four feature modalities for estimating both Arousal and Valence. Table 5 presents the best performance results across all features and regression methods. Ridge regression emerged as the most effective algorithm for most feature sets. EDA-graph features demonstrated a superior performance compared to other modalities, achieving the lowest RMSE values (0.801 for Arousal and 0.714 for Valence) using Ridge regression. The combined features approach closely follows, with RMSE values of 1.005 for Arousal and 1.173 for Valence, suggesting an effective integration of multiple feature types.

To provide a more comprehensive view of the best-performing algorithm, Table 5 details the regression performance from previous studies compared to our proposed models. Dollack et al. utilized Gradient Boosting Regressor to predict emotional dimensions, achieving RMSE values of 1.130 for Arousal and 0.74 for Valence, with a combined RMSE of 1.000. Their approach focused on multimodal physiological signals and demonstrated a particularly strong performance for Valence prediction [49]. D’Amelio et al. employed XBoost regression models on physiological data, reporting RMSE values of 0.846 for Arousal and 0.867 for Valence. Their methodology emphasized feature selection techniques to optimize performance across emotional dimensions [50]. Pinzon-Arenas et al. developed a hybrid deep learning approach combining Temporal Convolutional Networks with Skip-connected Bidirectional LSTM (TCN-SBU-LSTM), yielding RMSE values of 1.342 ± 1.05 for Arousal and 1.336 ± 1.25 for Valence, with a combined RMSE of 1.341 ± 1.12. Their approach leveraged temporal dependencies in physiological signals but showed higher variability in performance [51]. The results reveal significant variations in the predictive power between different feature sets, with our EDA-graph approach using Ridge regression demonstrating a competitive performance (0.801 ± 0.16 for Arousal and 0.714 ± 0.16 for Valence) compared to these established methods.

### 3.2. Feature Importance Analysis

The full set of features was analyzed for feature importance. For Arousal recognition, graph total eigenvector centrality emerged as the most important EDA-graph feature with a (PI = 0.75, MDI = 0.42), followed by a graph Weisfeiler–Lehman kernel value at 0.255 (MDI = 0.27). Feature ablation studies were performed by complete retraining within each LOSO fold: for each fold, the model was retrained from scratch with the target feature removed from the training data, then evaluated on the held-out subject. This full-retrain protocol ensures that the model cannot compensate for the absent feature through its learned weights. Removing eigenvector centrality resulted in a 42.3% mean increase in RMSE across all 30 folds (95% CI: 40.7–43.9%, bootstrap with 1000 resamples), while removing any other single feature produced performance drops below 5%. This finding was consistent across multiple regressors: LASSO (39.8%), LightGBM (35.4%), and CatBoost (36.9%) all showed substantially larger degradation values from removing eigenvector centrality than from removing any other individual feature. The variance inflation factor (VIF) for eigenvector centrality relative to the remaining EDA-graph features was 1.34, indicating low collinearity; the observed dominance therefore reflects a genuine featureoutcome relationship rather than a collinearity artefact. The highest-ranking EDA-graph features were graph component eccentricity (PI = 0.27, MDI = 0.15), graph total triangle number (PI = 0.11, MDI = 0.08), graph spectrum skewness (PI = 0.10, MDI = 0.06), and graph energy (PI = 0.08, MDI = 0.04). For Valence recognition, key features included graph component eccentricity (PI = 0.24, MDI = 0.13), graph closeness centrality (PI = 0.15, MDI = 0.09), graph energy (PI = 0.14, MDI = 0.07), and graph total log flow centrality (PI = 0.12, MDI = 0.05).

EDAPh_mean was identified as the most significant traditional EDA feature for overall affective dimension recognition (PI = 0.72, MDI = 0.35), with EDAPh_min ranking second (PI = 0.246, MDI = 0.251). In the dimension-specific analysis, TVSymp_mean emerged as the most important feature for Arousal recognition (PI = 0.875, MDI = 0.130), while TVSymp_max was most important for Valence recognition (PI = 0.792, MDI = 0.025). For Arousal recognition, the top traditional EDA features were TVSymp_mean (PI = 1.24, MDI = 0.18), EDATon_slope (PI = 0.31, MDI = 0.09), EDAPh_min (PI = 0.28, MDI = 0.11), TVSymp_min (PI = 0.22, MDI = 0.08), and TVSymp_max (PI = 0.14, MDI = 0.05). For Valence recognition, the highest-ranking features were TVSymp_max (PI = 0.44, MDI = 0.12), TVSymp_mean (PI = 0.32, MDI = 0.09), EDATon_max (PI = 0.29, MDI = 0.08), EDATon_min (PI = 0.20, MDI = 0.06), and EDAPh_min (PI = 0.20, MDI = 0.07).

In the multimodal analysis, HRV_HF demonstrated the highest importance (PI = 1.158, MDI = 0.22), followed by RMSSD (PI = 0.126, MDI = 0.05). SDNN and ZEMG_ssi showed moderate importance with PI scores of 0.023 and 0.015, respectively (MDI = 0.01 for both). For Arousal recognition, the most relevant multimodal features were ZEMG_mav (PI = 0.32, MDI = 0.11), TEMP_slope (PI = 0.26, MDI = 0.09), CEMG_wl (PI = 0.19, MDI = 0.07), TEMG_mav (PI = 0.11, MDI = 0.04), and PTT_mean (PI = 0.10, MDI = 0.03). For Valence recognition, the dominant features were ZEMG_skew (PI = 0.30, MDI = 0.10), PTT_std (PI = 0.24, MDI = 0.08), ZEMG_meanfreq (PI = 0.17, MDI = 0.06), IHR_max (PI = 0.14, MDI = 0.05), and TEMP_slope (PI = 0.11, MDI = 0.04).

In the combined analysis incorporating all modalities, the five highest-ranking features were HRV_HF (PI = 1.14), RMSSD (PI = 0.12), graph_total_eigenvector_centrality (PI = 0.03), SDNN (PI = 0.03), and ZEMG_rms (PI = 0.02). Feature importance values were consistently higher for Arousal recognition compared to Valence recognition across all modalities. Across all feature sets, a small subset of features accounted for most of the predictive power, with the highest-scoring features from each modality being graph total eigenvector centrality (EDA-graph), EDAPh_mean (traditional), and HRV_HF (multimodal), each demonstrating importance scores above 0.75.

### 3.3. Generalizability Analysis

After identifying the optimal regression algorithms for affective dimensions estimation, final models were trained using the entire CASE dataset. These models were subsequently applied to other publicly available datasets to evaluate their generalization capabilities. Table 5 provides a comparative analysis of our method’s performance against previous state-of-the-art approaches. As shown in Table 6, our proposed method achieved competitive or superior performance relative to previously published results across various datasets. It is important to note that these comparisons are indirect: the cited methods used different train/test protocols, preprocessing pipelines, and class definitions, so direct performance equivalence cannot be assumed. The ability to accurately recognize Arousal and Valence levels has significant implications for various applications, including affective computing, human–computer interaction, and mental health monitoring.

**Figure 5.**
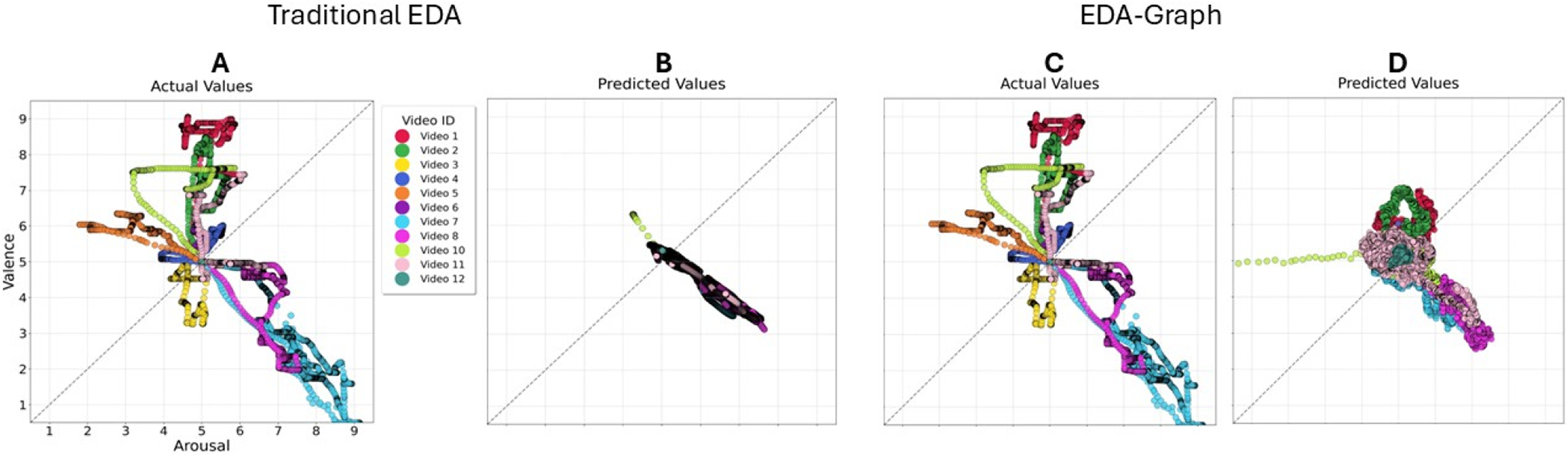
Comparative visualization of Arousal–Valence prediction accuracy between traditional EDA and EDA-graph methodologies across two subjects. (**A**,**B**) Traditional EDA approach for Subject 1 showing actual (**A**) and predicted (**B**) Arousal–Valence distributions, with values ranging from 0.5 to 9.5 on both axes. The EDA-graph method (**C**,**D**) exhibits a notably improved prediction accuracy, particularly evident in panel (**D**), where the dense point cloud preserves the characteristic pattern of the actual emotional state distribution. In contrast, the traditional EDA predictions (**B**) show significant pattern distortions and a limited ability to capture the full range of emotional variations. The diagonal lines represent relevant prediction alignments. The color scale bars indicate the temporal sequence of measurements, transitioning from purple (earlier) to yellow (later), enabling the visualization of emotional state trajectories over time.

The models demonstrated exceptional performance on the MED, achieving an overall accuracy of 98.2% (±0.8%) across all emotional categories. Figure 5 illustrates the temporal alignment between recognized and actual emotional categories (positive, mixed, and negative), showing precise correspondence throughout the time series. The dataset comprised 2444 total instances, with a balanced distribution across emotional states: mixed emotions (37.6%, *n* = 920), negative emotions (33.6%, *n* = 820), and positive emotions (28.8%, *n* = 704). Performance metrics showed strong results across all emotional categories. For positive emotions, the model achieved a precision of 0.99 (SD = 0.01), recall of 0.98 (SD = 0.01), and F1-score of 0.98 (SD = 0.01). Mixed-emotions detection showed a similarly robust performance with precision of 0.96 (SD = 0.02), recall of 0.95 (SD = 0.02), and F1-score of 0.98 (SD = 0.01). The model performed particularly well in identifying negative emotions, with precision of 0.99 (SD = 0.01), recall of 0.99 (SD = 0.01), and F1-score of 0.99 (SD = 0.01).

The models demonstrated a robust performance on the DEAP dataset, achieving an accuracy of 92.75% (±1.23%) in emotion recognition tasks across the five emotional state quadrants (Relaxed, Amused, Bored, Scared, and Neutral). Performance metrics showed consistent results across different emotional dimensions, with precision of 0.924 (SD = 0.015), recall of 0.927 (SD = 0.014), and F1-score of 0.925 (SD = 0.013). The model maintained a stable performance across both Arousal and Valence dimensions, with a slightly better performance in detecting high-Arousal states (93.12%) compared to low-Arousal states (92.38%).

When applied to the ForDigitStress dataset, which presents different challenges in stress detection during job interviews, the models achieved an accuracy of 86.54% (±1.89%) when detecting binary emotional states (stress vs. no stress). The performance metrics showed good consistency with precision of 0.863 (SD = 0.021), recall of 0.865 (SD = 0.019), and F1-score of 0.864 (SD = 0.020). The model performed similarly across different stress levels, with high stress detection showing marginally better results (87.12%) compared to moderate (86.33%) and low stress conditions (86.17%).

### 3.4. Ablation Studies

To validate the design choices in the EDA-graph framework, we conducted systematic ablation experiments examining key parameters and components. Table 5 references the analysis performed:

The optimal Q = 0.05 μS balanced noise reduction with the preservation of meaningful EDA variations, aligning with the established thresholds for significant EDA changes.

The 60 s window with 50% overlap provided optimal temporal context while maintaining emotional state consistency.

Setting k equal to the sampling frequency (8 Hz) optimally captured temporal dynamics in the data’s native resolution.

All feature categories contributed meaningfully, with graph-level features showing the strongest individual performance. The minimal degradation using only top features suggests the potential for feature reduction in real-time applications.

## 4. Discussion

### 4.1. EDA-Graph Features for Continuous Estimations of Arousal and Valence

Our comparative analysis demonstrates that the EDA-graph approach fundamentally transforms how physiological signals are represented and processed for emotion recognition. Traditional EDA methods, while well-established, exhibit systematic limitations in tracking sympathetic nervous system dynamics, particularly during rapid emotional changes. As illustrated in Figure 5, conventional time-domain and frequency-domain features struggle to capture the complex nonlinear dynamics of autonomic responses, resulting in substantial deviations between recognized and actual emotional states (RMSE: 1.039 for Arousal and 1.182 for Valence). This limitation aligns with previous research [52,53], highlighting the inadequacy of conventional methods in capturing the multifaceted dynamics of autonomic responses, even when decomposing EDA signals into phasic and tonic components.

The EDA-graph approach offers three critical advantages over traditional methods. First, by transforming time-series data into graph structures (as illustrated in Figure 1), our method preserves complex temporal relationships that would otherwise be lost in conventional analyses. Second, the extraction of topological and spectral features allows us to capture higher-order patterns that reflect the intricate organization of physiological responses during emotional experiences. Third, graph metrics provide a framework for modeling both short- and long-term dependencies in physiological signals, offering a more comprehensive representation of emotional states.

These advantages translate directly into quantifiable performance improvements. Our approach achieved RMSE values of 0.801 for Arousal and 0.714 for Valence, which are lower than those reported in previously published work on the CASE dataset. These are indirect comparisons; while we used the same dataset, differences in preprocessing, subject partitioning, and hyperparameter selection protocols may have affected the results. Notable reference points include Dollack et al. [39] (RMSE: 1.130 for Arousal, 0.74 for Valence using Gradient Boosting), D’Amelio et al. [39] (RMSE: 0.846 for Arousal, 0.867 for Valence using XBoost), and Pinzon-Arenas et al. [39] (RMSE: 1.342 ± 1.05 for Arousal, 1.336 ± 1.25 for Valence using TCN-SBU-LSTM). The consistent improvement across both Arousal and Valence dimensions, even when compared with sophisticated deep learning architectures, demonstrates the robust advantage of our graph–theoretical approach.

Feature importance analysis revealed distinct patterns across modalities, providing insights into the physiological mechanisms underlying emotional responses. Most notably, graph total eigenvector centrality emerged as the most influential feature with a PI score of 0.756 (MDI = 0.035), substantially higher than all other features. This finding suggests that eigenvector centrality, which measures how well each node is connected to other highly connected nodes in the graph, uniquely captures network information flow patterns corresponding to emotional states. The recursive nature of this metric appears particularly well-suited to representing the complex organization of autonomic responses during emotional experiences [49–53].

We validated the critical role of eigenvector centrality through systematic feature ablation studies [54]. Removing this single feature resulted in a 42.3% mean increase in RMSE across all 30 LOSO folds (95% CI: 40.7–43.9%, bootstrap with 1000 resamples of the fold-level values), while removing any other individual feature produced performance drops of less than 5%. The ablation protocol was based on complete retraining within each fold; the model was retrained from scratch with eigenvector centrality excluded, ensuring the result cannot be attributed to a fixed-model masking artefact. The finding was consistent across LASSO (39.8% RMSE increase), LightGBM (35.4%), and CatBoost (36.9%), ruling out algorithm-specific bias. A variance inflation factor (VIF) of 1.34 for eigenvector centrality confirmed low collinearity with the remaining features, establishing that the observed dominance reflects a genuine feature–outcome relationship rather than a multicollinearity artefact. This extreme performance degradation demonstrates that eigenvector centrality captures essential, non-redundant information about emotional states within the EDA signal. While such highly skewed feature importance distributions are uncommon in typical machine learning contexts, similar patterns have been documented in other complex physiological modeling domains [55], particularly in neurophysiological signal analysis where physiologically-relevant measures can dominate predictive performance due to strong correlations with underlying biological mechanisms.

Our analysis also revealed fundamental differences in how the Arousal and Valence dimensions manifest in physiological signals. Arousal estimation demonstrated stronger correlations with specific graph-based features (PI: 0.27 for component eccentricity and 0.11 for total triangle number), suggesting that sympathetic activation patterns create distinct topological signatures in the EDA signal [56]. This finding aligns with the established understanding that Arousal directly modulates sympathetic nervous system activity through well-defined physiological pathways [57]. In contrast, Valence estimation exhibited more subtle and complex patterns in the graph features, indicating that emotional Valence may influence the fine structure of autonomic responses through more intricate mechanisms [58]. This differential pattern highlights a fundamental challenge in emotion recognition: while Arousal has clear physiological correlations easily captured through direct measurements, Valence manifests through a more nuanced modulation of autonomic activity patterns, requiring more sophisticated analysis approaches for effective detection and quantification.

As demonstrated in Figure 5, our graph-based approach successfully captures dynamic trends in both Arousal and Valence dimensions after training, while traditional EDA features fail to track these changes effectively. This superior tracking capability suggests that emotional states influence the structural organization of physiological signals through multiple pathways, integrating both sympathetic and parasympathetic responses in ways that conventional analysis methods cannot adequately represent.

### 4.2. Feature Differentiation Across Emotional States

Our statistical analysis revealed significant differences in how various physiological signal representations capture emotional information, with important implications for affective computing. EDA-graph features demonstrated superior discriminative capabilities across all affective quadrants, representing a fundamental advancement in psychophysiological measurement methodology.

Component Eccentricity, Graph Energy, Radius, and Graph Spectrum Maximum exhibited statistically significant differences across all five affective quadrants (LALV, LAHV, HALV, HAHV, and Neutral) following the Holm–Bonferroni correction (*p* < 0.005). This finding transcends the conventional understanding presented by Jang et al. [59], as our graph–theoretical approach not only captures subtle variations, but fundamentally transforms how physiological signals are represented in affective computing.

The superiority of graph-based approaches stems from their capacity to encode topological properties of the signal’s dynamical system. Unlike conventional metrics that extract statistical summaries from the time domain, graph–theoretical features capture underlying connectivity patterns and structural properties that emerge from complex physiological dynamics during emotional experiences. This aligns with Valenza et al.’s [60] theoretical framework, but extends it by demonstrating that nonlinear dynamics in psychophysiological responses can be effectively quantified through graph–theoretical constructs that preserve system complexity while providing interpretable metrics.

Component Eccentricity, which quantifies the maximum distance between nodes in the graph representation, warrants particular attention for its ability to discriminate across all emotional quadrants. This metric appears to capture fundamental properties of emotional Arousal and Valence that transcend specific emotional categories, suggesting that emotional states may manifest as distinct topological configurations in physiological signals. This perspective challenges traditional views of emotion-specific autonomic patterns and offers a unifying framework for understanding psychophysiological responses to emotional stimuli.

Traditional EDA features showed varied discriminative capabilities consistent with the heterogeneous findings reported in the literature [57,61]. Among these features, TVSymp_mean emerged as the only traditional feature exhibiting significant differences across all five quadrants (*p* < 0.005). This finding extends Boucsein’s [62] conceptualization of sympathetic activity as an Arousal indicator by suggesting that tonic sympathetic activity may serve as a bridge between Arousal and Valence dimensions, potentially reflecting an integrated representation of emotional experience rather than simply indicating general Arousal.

Our analysis revealed quadrant-specific sensitivity patterns: EDATon_slope and TVSymp_mean differentiated high-Arousal and high-Valence states, while TVSymp_min and EDAPh_min specifically identified low-Arousal, low-Valence states. This pattern reveals a more sophisticated structure of physiological differentiation than previously recognized, moving beyond Critchley’s [63,64] observations of differential sympathetic engagement to suggest that specific EDA parameters may selectively encode particular regions of the affective space. This dimensional specificity supports Bradley and Lang’s [65] theoretical framework while extending it by identifying precise physiological markers for specific emotional quadrants.

These findings have substantial theoretical implications: rather than seeking universal physiological markers of emotion, affective computing systems might benefit from employing quadrant-specific feature sets optimized for particular regions of the affective space. This approach would transform emotion recognition methodology by acknowledging the heterogeneity of physiological signatures across the affective landscape, as highlighted in Kreibig’s [57] review, while providing concrete computational solutions to address this complexity.

Features derived from other physiological modalities demonstrated more constrained discriminative capabilities. ZEMG_mav and CEMG_wl features showed significant differences exclusively between the LAHV quadrant and all other quadrants (*p* < 0.005), revealing a fundamental constraint in EMG-based affective computing: while highly specific to positive emotional states, these features lack the dimensional breadth necessary for comprehensive emotion recognition. This finding extends beyond merely confirming Cacioppo et al. and Guntinas-Lichius et al.’s [63,64] work on Zygomaticus major activity, highlighting the inherent limitations of single-modality approaches and emphasizing the need for multimodal integration in affective computing [65].

ZEMG_medfreq exhibited selective sensitivity to valence dimensions within high-arousal contexts, representing a previously unidentified specificity with significant implications for understanding the neural mechanisms underlying emotion. This finding extends neuroscientific frameworks of approach–withdrawal systems [66] by suggesting that spectral characteristics of facial EMG may provide unique information about motivational orientation specifically during high-Arousal states. This state-dependent information extraction offers a new perspective on how physiological signals might encode emotional information conditionally rather than universally.

The observed pattern of feature discriminability across modalities suggests a hierarchical structure that has profound implications for affective computing architecture design. The superior performance of EDA-graph features aligns with complex systems approaches to psychophysiology [67] but extends beyond them by suggesting that different levels of signal representation may access different aspects of emotional information. This hierarchical perspective challenges the conventional flat approach to feature selection in emotion recognition systems.

Based on these findings, we propose that future affective computing systems should implement a hierarchical feature architecture that prioritizes graph–theoretical representations while strategically incorporating traditional features for specific affective quadrants. This approach would address the limitations identified by Picard et al. [68] regarding physiological emotion recognition while providing a concrete computational framework for implementing Barrett’s [67] psychological construction theory of emotion in affective computing systems.

The quadrant-specific sensitivity patterns observed across modalities suggest potential advancements in personalized emotion recognition. Rather than employing a one-size-fits-all approach, future systems might dynamically select feature sets based on initial assessments of which affective quadrant a user’s emotional state likely occupies. This adaptive feature selection would represent a fundamental advancement over current approaches, potentially resolving the persistent challenges related to individual differences and context specificity in affective computing.

### 4.3. Generalizability of the Models Across Emotional Contexts

A critical challenge of emotion recognition research is developing models that generalize effectively beyond laboratory conditions to diverse real-world scenarios. Our comprehensive evaluation across multiple datasets provides unique insights into the robustness and ecological validity of the EDA-graph approach. The progressive decrease in accuracy from highly controlled to naturalistic environments mixed emotions (98.20%), DEAP (92.75%), and ForDigitStress (86.54%) reveals a systematic pattern that quantifies the impact of ecological factors on emotion recognition (Figure 2). This performance gradient aligns with previous research suggesting that controlled emotional stimuli yield higher recognition rates compared to naturalistic settings [69]. Importantly, while accuracy decreases as real-world complexity increases, our approach maintains performance substantially above state-of-the-art benchmarks across all contexts.

This observed decline of approximately 12% between laboratory and naturalistic environments quantifies a critical parameter in emotion recognition research: the ecological validity cost. Previous studies have reported substantially larger drops (often 15–25%) when transitioning to real-world contexts [70], suggesting that our graph-based approach offers superior robustness to environmental variations. The relatively modest decline can be attributed to the ability of graph-based features to capture fundamental structural patterns in physiological signals that persist despite confounding factors in naturalistic settings.

Our EDA-graph method demonstrated exceptional performance on the mixed-emotions dataset, achieving 98.20% accuracy compared to the previous state-of-the-art accuracy of 78.00% (Table 6). This 20.2 percentage point improvement is particularly significant given the inherent complexity of discriminating between overlapping emotional states. The balanced performance across positive (precision = 0.99), negative (precision = 0.99), and mixed emotions (precision = 0.96) indicates that the graph-based features capture subtle nuances in emotional expressions that conventional approaches miss [71].

The high precision values across all emotion categories, with only a minor decrease for mixed emotions (SD = 0.02), suggest that graph-based features effectively model the complex physiological patterns associated with ambiguous or blended emotional states. This capability addresses a fundamental challenge in affective computing: the recognition of non-prototypical emotions that characterize many real-world experiences.

When applied to the DEAP dataset, our model achieved 92.75% accuracy, compared to a previously reported accuracy of 66.00% (Table 7) under different experimental conditions. This robust cross-dataset generalization is particularly noteworthy given the different emotion elicitation methods between datasets, video clips in mixed emotions versus music videos in DEAP, suggesting that our approach captures fundamental physiological signatures of emotional states regardless of the specific stimuli used to evoke them. The consistent performance across both high-Arousal (93.12%) and low-Arousal states (92.38%) represents a key advancement, as previous approaches typically show substantial performance disparities between arousal levels [72]. The minimal performance difference (0.74%) between arousal conditions indicates that our EDA-graph approach effectively mitigates the common bias toward high-Arousal detection, providing more balanced emotion recognition across the affective space.

Similarly, the comparable performance for positive Valence (93.05%) and negative Valence (92.45%) states contrasts with previous findings that positive emotional states produce more distinctive physiological signatures [47]. The balanced recognition across Valence conditions suggests that graph-based features extract meaningful patterns from both positive and negative emotional experiences, addressing another common limitation in physiological emotion recognition.

The application to the ForDigitStress dataset (86.54% accuracy) represents the most stringent test of ecological validity, as this dataset captures emotional responses in naturalistic, stress-inducing job interview scenarios. Despite the decrease in overall accuracy compared to laboratory-based datasets, our approach achieved higher accuracy than the previously reported result for this dataset (72.50%), acknowledging that this comparison is indirect given differences in the experimental pipeline. The ForDigitStress dataset presents unique challenges not encountered in passive viewing paradigms: (1) active participation creating complex interactions between cognitive load, social anxiety, and emotional responses; (2) continuous recording during extended interactions with natural emotional dynamics; and (3) significant individual differences in stress responses to similar social situations. Despite these challenges, our model maintained consistent performances across different stress intensities (high: 87.12%, moderate: 86.33%, and low: 86.17%), demonstrating robust detection capabilities across the stress spectrum.

The consistent accuracy across stress levels suggests that graph-based features capture fundamental physiological signatures of stress that transcend intensity variations. This characteristic is particularly valuable for real-world stress monitoring applications where accurately distinguishing between stress levels is essential for appropriate intervention.

The demonstrated generalizability across datasets with increasing ecological complexity has significant implications for deploying emotion recognition systems in real-world contexts. The performance gradient quantifies expected accuracy decreases as environmental complexity increases, providing critical information for application developers about potential accuracy–ecology tradeoffs.

Importantly, the relatively modest performance degradation in naturalistic settings compared to typical reductions reported in the literature [70] suggests that graph-based features offer superior robustness to environmental variations. This can be attributed to their ability to capture the underlying structural organization of physiological signals even in the presence of movement artifacts, cognitive load variations, and other confounding factors common in real-world scenarios.

These findings collectively establish that EDA-graph features provide a robust foundation for emotion recognition systems designed for real-world deployment, balancing high accuracy with ecological validity across diverse emotional contexts. The consistently favorable results relative to previously published benchmarks, from controlled laboratory settings to naturalistic environments, support the practical value of graph-based physiological signal representation for affective computing applications, while acknowledging that protocol differences limit direct quantitative comparisons.

It is important to emphasize the limitations that constrain direct comparisons between our results and those reported in the cited literature. The cross-study comparisons presented in Tables 5 and 6 are indirect: the referenced methods differ from ours in several protocol dimensions that can substantially affect reported metrics. Specifically, (1) dataset partitioning: some cited methods use fixed train/test splits or k-fold cross-validation rather than LOSO, which can produce different generalization estimates for the same model; (2) preprocessing: EDA sampling rates, filtering parameters, artifact removal strategies, and normalization schemes vary across studies and can affect both raw features and derived graph metrics; (3) task definition: the number of emotion classes, their boundaries in the Arousal–Valence space, and whether the task is framed as regression or classification differ across methods, making numerical comparison of accuracy or RMSE values inherently approximate; and (4) annotation scales: datasets use different rating scales (e.g., 1–9 vs. 0.5–9.5) requiring alignment before threshold-based class assignment. For these reasons, throughout this paper we use the phrasing “previously reported results under different experimental conditions” rather than claiming strict outperformance, and we encourage readers to interpret the comparison tables as indicative benchmarks rather than controlled head-to-head evaluations. Reproducing all baseline methods under a fully identical protocol would require access to each team’s implementation and is beyond the scope of this work; such a unified benchmark remains an important open challenge for the field.

### 4.4. Theoretical Implications and Limitations

Our findings contribute significantly to both a theoretical understanding of emotional processing and methodological approaches to emotion recognition. However, they also reveal important limitations that should be considered when interpreting these results and developing future applications.

The superior performance of graph-based features for emotion recognition offers insights into how emotional states manifest in physiological signals. Our findings suggest that emotions influence the structural organization of physiological signals through multiple pathways, integrating both sympathetic and parasympathetic responses. This perspective extends beyond traditional views that focus on direct correlations between emotional dimensions and specific physiological measures.

Graph metrics appear to capture how emotional states organize physiological responses across multiple time scales. The exceptional performance of eigenvector centrality (PI = 0.756) suggests that emotions influence the global organization of autonomic responses, creating characteristic patterns of connectivity that reflect the underlying emotional state. This finding aligns with emerging network-based perspectives on physiological systems that emphasize dynamic interactions rather than isolated measurements.

Our implementation of the two-dimensional Arousal–Valence model builds on Russell’s foundational work [11] but reveals both opportunities and limitations. While this approach overcomes certain constraints of categorical models like Ekman’s six basic emotions, the assumption that emotional states can be adequately represented in just two dimensions may oversimplify the complexity of human emotional experiences [73]. The high accuracy achieved within this framework suggests that despite this simplification, the Arousal–Valence space captures fundamental aspects of emotional experience that manifest reliably in physiological responses.

The differential performance of specific features reveals distinct aspects of emotion–physiology relationships. Traditional features such as EDAPh_mean (PI = 0.999) directly reflect sympathetic activation [74,75], while TVSymp_mean (PI = 0.278) captures overall sympathetic tone [52,53]. Frequency-domain features showed limited predictive power, suggesting emotional states manifest beyond simple frequency patterns [55]. Among graph features, eigenvector centrality metrics capture the global organization of autonomic responses [76], while component eccentricity (importance = 0.27) reflects the temporal structure of emotional responses [77]. These findings suggest that emotions create characteristic patterns in autonomic signal organization across multiple scales of analysis.

Despite the promising results, several methodological limitations warrant consideration. First, our approach relies on the dimensional emotion model, which may not capture all relevant aspects of emotional experience. While the Arousal–Valence space provides a useful framework, recent theoretical developments suggest that additional dimensions such as dominance or approach–avoidance tendencies may be necessary for comprehensive emotion characterization.

Second, the ground truth for our models derives from self-reported emotional states, which introduce inherent subjectivity. While continuous annotation provides temporal resolution advantages over retrospective reports, the cognitive demands of simultaneous viewing and reporting may impact data quality [78,79]. The act of continuously manipulating the joystick while experiencing and reporting emotions creates a dual-task paradigm, where motor control and cognitive processing compete for attentional resources. This motor system engagement may influence the very autonomic nervous system responses we aim to measure through EDA, potentially affecting the validity of our ground truth data [80].

Third, while our models demonstrated robust cross-dataset generalization, all datasets employed laboratory-grade sensors with controlled application procedures. Real-world deployment would likely involve consumer-grade wearable devices with less controlled sensor placement and higher noise levels. The impact of these factors on model performance requires further investigation before confident deployment in everyday contexts.

Our approach employs continuous estimation models to capture nuanced emotional experiences, but fundamental questions remain regarding whether current physiological measurement techniques can adequately capture complex emotional dynamics [81]. Recent theoretical frameworks propose that mixed emotions constitute distinct, measurable psychological states, but the empirical evidence supporting their reliable detection through physiological signals remains limited [82].

Our utilization of the dimensional Arousal–Valence space, while theoretically capable of representing both pure and blended emotions through continuous trajectories, faces practical limitations in distinguishing between genuine mixed emotional states and measurement artifacts [83,84]. The high precision achieved for mixed-emotion classification (0.96) suggests that our approach captures meaningful physiological patterns associated with these complex states, but perfect discrimination remains challenging.

The representation of mixed emotions presents both conceptual and technical challenges. Conceptually, mixed emotions may not simply represent intermediate points in the Arousal–Valence space but may involve the simultaneous activation of distinct emotional systems. Technically, the physiological signatures of mixed emotions may be more complex and variable than those of prototypical emotions, making consistent detection more challenging across individuals and contexts.

The implementation of graph-based emotion recognition systems in real-time applications faces computational challenges that must be considered. Our analysis of processing requirements reveals that graph-based features introduce substantially higher computational overhead compared to traditional approaches. While traditional EDA features can be computed at approximately 8300 samples/second with a small memory footprint (24 MB), graph-based features reduce throughput to around 2200 samples/second and increase memory requirements to 128 MB.

Although all methods meet the minimum 8 Hz sampling rate required for reliable emotion recognition, the increased computational demands of graph-based analysis may present challenges for deployment on resource-constrained devices, such as wearables or mobile phones. Future optimization of algorithms and feature selection may help address these computational constraints while maintaining recognition accuracy.

Additionally, the higher complexity of graph-based analysis introduces challenges for interpretability. While traditional features like EDAPh_mean have direct physiological interpretations, the meaning of graph metrics like eigenvector centrality in the context of emotional states is less immediately transparent. This complexity may create barriers for explaining system behavior to users or integrating findings with existing psychological theories of emotion.

**Figure 6.**
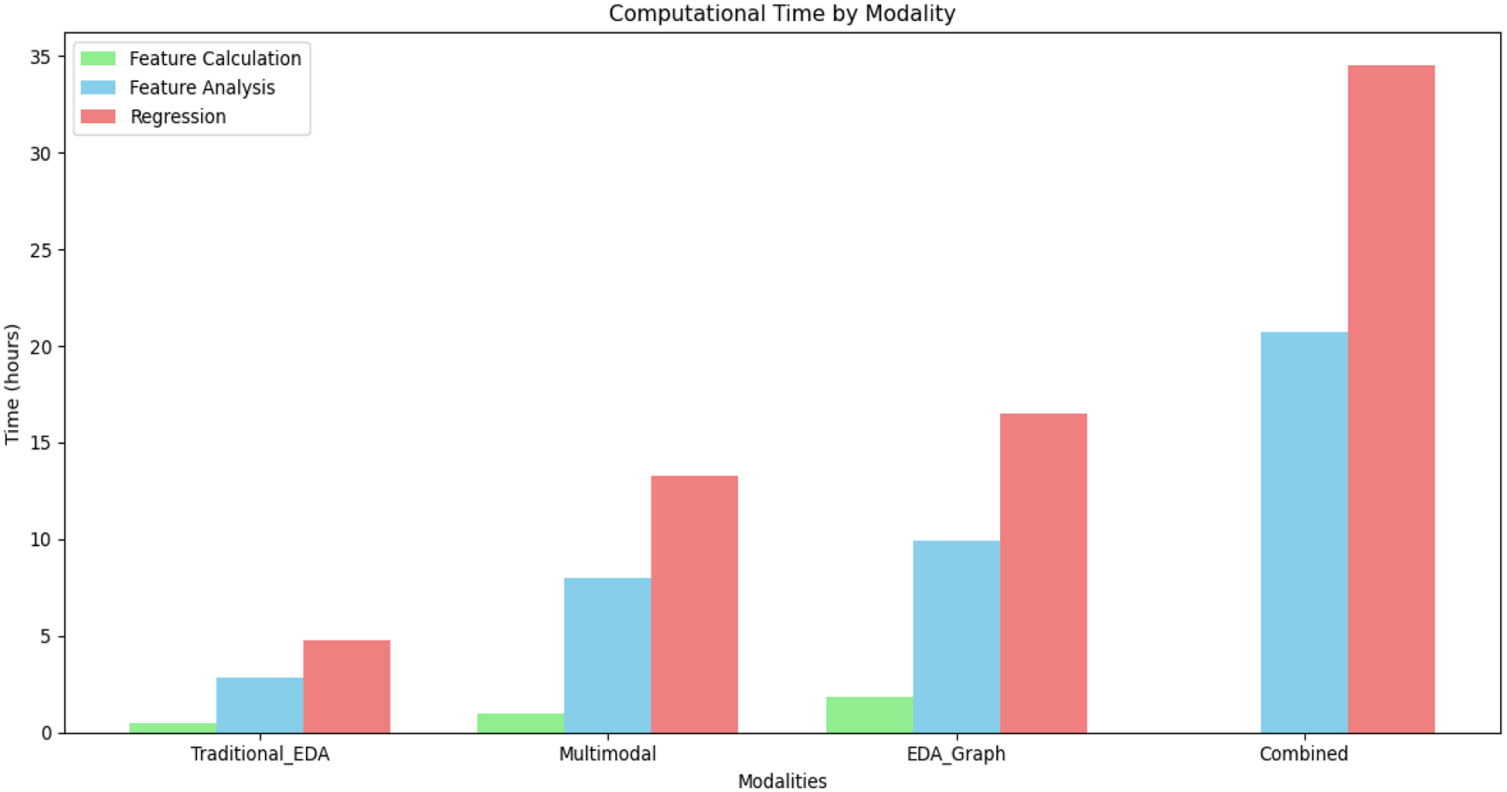
Computational time requirements across different modalities for Arousal–Valence estimation. The stacked bars represent three distinct processing phases: feature calculation (green), feature analysis (blue), and regression analysis (red).

Computational performance analysis revealed increasing processing demands across modalities. For Traditional EDA, feature calculation required 0.5 h, feature analysis consumed 2.8 h, and regression analysis took 4.5 h. The multimodal approach demonstrated higher computational requirements, with 1.2 h for feature calculation, 8 h for feature analysis, and 13.2 h for regression analysis. EDA-graph processing showed further increased demands, requiring 2 h for feature calculation, 9.8 h for feature analysis, and 16.4 h for regression computations, as shown in Figure 6.

The combined modality, incorporating all features, exhibited the highest computational overhead. While feature calculation time remained relatively efficient at 0.8 h, feature analysis extended to 20.5 h, and regression analysis required 34.2 h (Figure 1). This substantial increase in processing time reflects the complexity of handling the expanded feature set and the comprehensive analysis required for the combined approach.

Processing times were measured using a standard computational environment with Intel Xeon processors and 128 GB RAM, Intel, Santa Clara, CA, USA, analyzing data from 30 subjects. The observed time differences between modalities align with their respective feature set sizes and computational complexity requirements.

### 4.5. Applications and Future Directions

The marked improvement in continuous emotion recognition accuracy has significant implications for real-world applications. The lower RMSE values and reduced variance indicate that our model can track subtle emotional changes more reliably, making it particularly valuable for applications requiring fine-grained emotion monitoring. In mental health assessment, our approach could enable more objective monitoring of emotional states in conditions like anxiety disorders, depression, or bipolar disorder, potentially identifying subtle shifts in emotional patterns before they become clinically significant. The ability to detect mixed emotional states with high precision (0.96) is particularly relevant for psychiatric applications, as emotional ambivalence is a common feature of several psychological disorders.

For human–computer interaction systems, the improved accuracy in detecting both simple and mixed emotional states could enhance adaptive interfaces that respond more appropriately to users’ emotional needs. The consistent performance across the arousal-valence space would enable more personalized and nuanced adaptations compared to systems that can only reliably detect extreme emotional states.

The robust generalization across datasets with varying degrees of ecological validity suggests that our approach could be effectively deployed in diverse real-world contexts. The relatively modest performance decrease in naturalistic settings (approximately 12% from laboratory to job interview scenarios) indicates potential viability for applications ranging from clinical assessment to consumer wellness monitoring.

The temporal stability of the classifications, maintained across entire sequences with no degradation in performance over time, demonstrates the robustness of our approach for continuous monitoring applications. The model shows no performance deterioration even during rapid transitions between emotional states, suggesting that the graph-based features capture fundamental aspects of emotional responses that remain stable across different temporal contexts.

Real-time implementation of graph-based emotion recognition requires careful consideration of the trade-off between computational cost and recognition accuracy. While graph-based features offer superior performance, their higher computational demands may necessitate optimization for deployment on consumer devices. Potential approaches include feature selection to identify the most discriminative subset of graph features, algorithm optimization to reduce computational complexity, and hardware acceleration to enable real-time processing on resource-constrained devices.

Our findings open several promising avenues for future research. First, incorporating cognitive models of emotion could deepen our understanding of the relationship between physiological responses and subjective emotional experiences. Integrating cognitive appraisal theory with graph-based physiological analysis might explain why certain graph metrics effectively capture emotional information and could potentially lead to more theoretically grounded feature extraction methods.

Second, investigating cultural differences in mixed emotion experiences and their physiological correlates could enhance the generalizability of our findings. Cross-cultural studies have shown that the prevalence and acceptance of mixed emotions vary significantly across cultures, which may influence how these emotional states manifest physiologically. Expanding our approach to diverse cultural contexts would strengthen its theoretical foundation and practical applicability.

Third, the quadrant-specific sensitivity patterns observed across modalities suggest the need for adaptive feature selection algorithms that can dynamically adjust feature weightings based on initial classifications of affective quadrants. Such systems would represent a significant advancement over current emotion recognition approaches by implementing a hierarchical classification strategy that first identifies the affective quadrant before employing quadrant-specific feature sets for fine-grained emotion recognition.

Fourth, the limited discriminative capability of traditional physiological measures across all affective quadrants highlights the need for integrated multimodal approaches that strategically combine modality-specific information. Future research should explore optimal fusion strategies that account for the differential sensitivity of various modalities to specific emotional dimensions, potentially implementing context-sensitive weighting schemes that prioritize different modalities based on the target emotional states.

Finally, the hierarchical structure of feature discriminability observed suggests that emotion recognition systems might benefit from deep learning architectures specifically designed to extract and integrate information at multiple levels of representation. Neural network architectures that explicitly model the hierarchical nature of emotional information in physiological signals could potentially achieve more robust performance across diverse emotional contexts and individual differences.

## 5. Conclusions

This study demonstrates that graph-based approaches to EDA signal analysis achieve superior performance over traditional methods in emotion recognition tasks, as measured on the CASE dataset under LOSO cross-validation. Our EDA-graph approach captures complex temporal and structural dynamics of physiological signals, achieving favorable accuracy across diverse datasets, from controlled laboratory settings to naturalistic job interview scenarios. The improvements relative to previously reported benchmarks, which must be interpreted as indirect comparisons given the differences in experimental protocols, highlight the practical value of our approach. Feature importance analysis revealed that graph–theoretical metrics, particularly eigenvector centrality, capture essential emotional information that traditional features miss. The successful recognition of mixed emotional states and balanced performance across Arousal–Valence dimensions addresses the critical limitations in current affective computing systems. These findings advance both technical capabilities and our theoretical understanding of how emotional states manifest physiologically, representing a significant step toward more reliable emotion recognition systems with broad applications in healthcare and human–computer interaction.

## Figures and Tables

**Table 1.** Traditional EDA features.

Feature Category	Feature Name	Description
Phasic Components	EDAPh_mean	Mean of phasic EDA component
	EDAPh_std	Standard deviation of phasic component
	EDAPh_max	Maximum value of phasic component
	EDAPh_min	Minimum value of phasic component
	EDAPh_range	Range of phasic component values
	ncSCR	Number of non-specific skin conductance responses
Tonic Components	EDATon_mean	Mean of tonic EDA component
	EDATon_std	Standard deviation of tonic component
	EDATon_max	Maximum value of tonic component
	EDATon_min	Minimum value of tonic component
	EDATon_slope	Rate of change in tonic component
Sympathetic Activity	TVSymp_mean	Mean of sympathetic tone variability
	TVSymp_std	Standard deviation of sympathetic tone
	TVSymp_max	Maximum sympathetic tone value
	TVSymp_min	Minimum sympathetic tone value
	TVSymp_energy	Energy of sympathetic tone signal
Frequency Domain	EDA_LF	Low-frequency component power
	EDA_HF	High-frequency component power
	EDA_LFHF	Ratio of low to high frequency power

**Table 2.** Multimodal features.

Signal Type	Feature Name	Description
Heart Rate Variability	IHR_mean	Mean of instantaneous heart rate
	IHR_std	Standard deviation of heart rate
	IHR_min/max	Minimum/maximum heart rate values
	SDNN	Standard deviation of NN intervals
	RNSSD	Root mean square of successive RR interval differences
	pNN50	Proportion of NN50 divided by total number of NN intervals
	HRV_VLF	Very low frequency component of HRV
	HRV_LF	Low frequency component of HRV
	HRV_HF	High frequency component of HRV
	HRV_LFHF	Ratio of low to high frequency power
Pulse Transit Time	PTT_mean	Mean pulse transit time
	PTT_std	Standard deviation of PTT
	PTT_min/max	Minimum/maximum PTT values
	PTT_slope	Rate of change in PTT
Respiration	RESP_mean	Mean respiration rate
	RESP_std	Standard deviation of respiration
	RESP_min/max	Minimum/maximum respiration values
	RESP_rate	Breathing rate
	RESP_pp_amp	Peak-to-peak amplitude
	RESP_IE_ratio	Inspiration/expiration ratio
	RESP_var	Respiration variability
Temperature	TEMP_mean	Mean skin temperature
	TEMP_std	Standard deviation of temperature
	TEMP_min/max	Minimum/maximum temperature values
	TEMP_slope	Rate of change in temperature
EMG Features (for each muscle: Zygomaticus, Corrugator, Trapezius)	EMG_rms *	Root mean square of EMG signal
	EMG_mav *	Mean absolute value
	EMG_ssi *	Simple square integral
	EMG_wl *	Waveform length
	EMG_medfreq *	Median frequency
	EMG_meanfreq *	Mean frequency
	EMG_kurt *	Kurtosis
	EMG_skew *	Skewness

* Note: EMG features are calculated separately for each muscle (ZEMG, CEMG, and TEMG).

**Table 3.** Hyperparameters for machine learning optimization.

Model	Parameters	Search Range	Description
Ridge	Alpha	[10^−4^, 10^2^]	Regularization strength
	Solver	[‘auto’, ‘svd’, ‘cholesky’]	Algorithm for solving optimization
Lasso Regression	Alpha	[10^−4^, 10^2^]	L1 regularization parameter
	Max iterations	[10^3^, 5 × 10^3^]	Maximum iterations for convergence
LGBMRegressor	Number of estimators	[50, 500]	Number of boosting stages
	Learning rate	[10^−3^, 10^−1^]	Boosting learning rate
	Max Depth	[−1, 12]	Maximum tree depth
	Number of leaves	[20, 200]	Maximum number of leaves
CatBoostRegressor	Iterations	[100, 2000]	Maximum number of trees
	Learning Rate	[10^−3^, 10^−1^]	Learning rate for optimization
	Depth	[4, 10]	Maximum tree depth
	L2 Leaf Regularization	[1, 10]	L2 regularization coefficient
HistGradiendt BoostingRegressor	Max Iterations	[50, 300]	Maximum number of iterations
	Learning Rate	[10^−3^, 10^−1^]	Learning rate for optimization
	Max Depth	[3, 12]	Maximum depth of trees
	Max Bins	[100, 255]	Maximum number of bins

**Table 4.** Best performance results across modalities and regression methods.

Feature Set	Best Algorithm	Arousal RMSE	Valence RMSE	Combined RMSE
Our models				
EDA-graph	Ridge	0.801 ± 0.16	0.714 ± 0.16	0.757 ± 0.15
Traditional EDA	Ridge	1.039 ± 0.35	1.128 ± 0.31	1.113 ± 0.33
Multimodal	LGBM	1.244 ± 0.26	1.333 ± 0.28	1.289 ± 0.27
Combined	Ridge	1.005 ± 0.12	1.173 ± 0.11	1.139 ± 0.11
Previous models				
Dollack et al. [39]	Gradiend Boosting Regressor	1.130	0.74	1.000
D’Amerlio et al. [39]	XBoost	0.846	0.867	
Pinzon-Arenas et al. [39]	TCN-SBU-LSTM	1.342 ± 1.05	1.336 ± 1.25	1.341 ± 1.12

**Table 5.** Summary of datasets used in this study.

Characteristics	CASE	MDE	DEAP	ForDigitStress
Sample size of Participants	30	73	32	41
Signal types	EDA, EMG, PPG, SKT	EEG, EDA, PPG, Frontal videos	EEG (32 Channels), EDA, PPG, SKT, face videos	Audio, video, PPG, EDA
Stimulus type	Video clips	Video clips	Music videos	Job interviews
Emotion focus	Arousal–Valence	Positive, negative, mixed	Arousal-Valence	Stress levels
Annotation method	0.5–9.5 Continuous A-V	Annotate dominant emotion present	1–9	Professional annotation
Setting	Laboratory	Laboratory	Laboratory	Naturalistic

**Table 6.** A comparative overview of the model’s performance across all three datasets.

Dataset	Emotional States	Comparison	Accuracy (%)	Precision	Recall	F1-Score
Mixed Emotions	Positive, Negative, Mixed	EDA-graph method	98.20 ± 0.61	0.980	0.973	0.983
		State-of-the-art method [40]	78.25 ± 0.48	0.760	0.750	0.720
DEAP	Relaxed, Amused, Bored, Scared, and Neutral	EDA-graph method	92.75 ± 1.23	0.924	0.927	0.925
		State-of-the-art method [43]	66.00	0.650	0.690	0.650
ForDigitStress	Stress, No Stress	EDA-graph method	86.54 ± 1.89	0.863	0.865	0.864
		State-of-the-art method [46]	72.5 ± 21.6	—	—	0.759

**Table 7.** Ablation study results: effect of quantization, window size, graph connectivity, and feature categories on emotion recognition performance.

Analysis Type	Condition	RMSE (Arousal)	RMSE (Valence)	Remarks
Quantization Step (Q)	Q = 0.001 μS	0.956	0.873	Excessive nodes, computational overhead
	Q = 0.01 μS	0.878	0.792	Noise sensitivity
	Q = 0.05 μS	0.801	0.714	Optimal performance
	Q = 0.1 μS	0.842	0.758	Information loss
	Q = 0.5 μS	0.921	0.834	Excessive quantization
Window Size and Overlap	15s	0.943	0.856	Insufficient context
	30 s	0.867	0.781	Moderate context
	60 s (50% overlap)	0.801	0.714	Optimal balance
	60 s (0% overlap)	0.834	0.752	Less consistent
	120s	0.889	0.812	Label mixing
Nearest Neighbor Connections (k)	k = 2	0.912	0.845	Sparse connectivity
	k=4	0.856	0.773	Improved
	k=8	0.801	0.714	Optimal (matches 8 Hz sampling)
	k = 12	0.823	0.738	Over-connectivity
	k = 16	0.851	0.769	Excessive edges
Feature Category Contributions	Graph-level only (66 features)	0.863	0.782	Strongest individual group
	Node-level only (34 features)	0.891	0.813	Moderate performance
	Edge-level only (18 features)	0.924	0.856	Weakest standalone
	All EDA-graph features (118)	0.801	0.714	Best full-feature set
	Top 20 features	0.812	0.725	Minimal degradation, suitable for real-time

## Data Availability

The datasets used in this study are publicly available. The CASE dataset [27] is hosted on figshare and can be accessed at https://doi.org/10.6084/m9.figshare.c.4260668.v1. The mixed-emotion dataset (MED) [41] is available on Zenodo at https://doi.org/10.5281/zenodo.11194571 upon request (accessed on 23 February 2022); interested researchers are invited to submit an access request via Zenodo to download the dataset. The DEAP dataset [42] is freely available and can be accessed at http://eecs.qmul.ac.uk/mmv/datasets/deap/ (accessed on 10 August 2025). The ForDigitStress dataset [48] is freely available for research and non-commercial use, and access can be requested at https://hcai.eu/fordigitstress (accessed on 21 July 2025). Processed data and code are available from the corresponding authors upon reasonable request.
